# A systematic review on the link between adverse childhood experiences (ACE) and later involvement in gang violence and extremist groups

**DOI:** 10.1192/j.eurpsy.2023.650

**Published:** 2023-07-19

**Authors:** I.-C. Matei

**Affiliations:** MSc Global Mental Health, University of Glasgow, Glasgow, United Kingdom

## Abstract

**Introduction:**

Adverse childhood experiences are common and have been linked to a number of physical illnesses, as well as socioeconomic problems. Moreover, it has been proven that ACEs can increase chances of people showing criminal behaviour. The question arises whether people with ACEs also have an increased chance of joining extremist groups or violent gangs.

**Objectives:**

The aim of this systematic review is to measure the ACE rates in violent extremists and gangs and to establish whether there is a pattern linking ACEs to violent extremist organizations and gangs.

**Methods:**

The following databases were searched to retrieve relevant studies**:** the ProQuest Social Science database, Pubmed, Scopus. Eligible studies were articles of any study design that reported ACE rates in either extremists or gang members. Data was extracted and organized into a table and a quality assessment was performed using standardized tools (CASP and NHLBI). A narrative synthesis of the evidence was conducted. A meta-analysis could not be performed due to the heterogeneity of the studies.

**Results:**

22 studies (eight on extremists and fourteen on gang members) were included. The studies varied in terms of research design, sample size, location and measured ACEs. Quality also varied across the studies. The prevalence rates were heterogenous and ranged from 0% to almost 100%.

Physical abuse was the most addressed ACE (5 studies on extremists and 11 on gang members). Sexual abuse was the second most explored ACE (4 studies on extremists and 9 studies on gang members). Neglect and caregiver loss were also common ACEs, while the other ACEs were less represented in the two subpopulations. A comparison between the two subpopulations was difficult due to the differences in the studies.

**Conclusions:**

While overall ACE rates were high in the two groups and some ACEs were salient in the two subpopulations, quality of evidence varied across the studies. No solid ACE pattern across the studies could be found. Moreover, there were only two prospective studies on gang members and none on extremists, so a causal relationship between ACEs and involvement in violent gangs or extremist organizations could not be established. Future research should concentrate on studies of this design, as well as on improving the quality of the evidence.

As ACEs are extremely common, researchers should also look beyond them when searching for causes of extremism or violent gang membership. Other negative events (bullying, racism) should also be explored.

**Disclosure of Interest:**

None Declared

